# APOBEC-induced mutations and their cancer effect size in head and neck squamous cell carcinoma

**DOI:** 10.1038/s41388-018-0657-6

**Published:** 2019-01-15

**Authors:** Vincent L. Cannataro, Stephen G. Gaffney, Tomoaki Sasaki, Natalia Issaeva, Nicholas K. S. Grewal, Jennifer R. Grandis, Wendell G. Yarbrough, Barbara Burtness, Karen S. Anderson, Jeffrey P. Townsend

**Affiliations:** 1 Department of Biostatistics, Yale School of Public Health, New Haven, CT USA; 2 Department of Pharmacology, Yale University, New Haven, CT USA; 3 Yale Cancer Center, Yale University, New Haven, CT USA; 4 Division of Otolaryngology, Department of Surgery, Yale School of Medicine, New Haven, CT USA; 5 Department of Ecology and Evolutionary Biology, Yale University, New Haven, CT USA; 6 Department of Otolaryngology-Head and Neck Surgery, University of California San Francisco, San Francisco, CA USA; 7 Department of Pathology, Yale School of Medicine, Yale University, New Haven, CT USA; 8 Department of Medicine, Yale School of Medicine, Yale University, New Haven, CT USA; 9 Department of Molecular Biophysics & Biochemistry, Yale University, New Haven, CT USA; 10 Program in Computational Biology and Bioinformatics, Yale University, New Haven, CT USA

**Keywords:** Oncogenes, Tumour virus infections, Head and neck cancer, Cancer genomics, Cancer genetics

## Abstract

Recent studies have revealed the mutational signatures underlying the somatic evolution of cancer, and the prevalences of associated somatic genetic variants. Here we estimate the intensity of positive selection that drives mutations to high frequency in tumors, yielding higher prevalences than expected on the basis of mutation and neutral drift alone. We apply this approach to a sample of 525 head and neck squamous cell carcinoma exomes, producing a rank-ordered list of gene variants by selection intensity. Our results illustrate the complementarity of calculating the intensity of selection on mutations along with tallying the prevalence of individual substitutions in cancer: while many of the most prevalently-altered genes were heavily selected, their relative importance to the cancer phenotype differs from their prevalence and from their *P* value, with some infrequent variants exhibiting evidence of strong positive selection. Furthermore, we extend our analysis of effect size by quantifying the degree to which mutational processes (such as APOBEC mutagenesis) contributes mutations that are highly selected, driving head and neck squamous cell carcinoma. We calculate the substitutions caused by APOBEC mutagenesis that make the greatest contribution to cancer phenotype among patients. Lastly, we demonstrate via in vitro biochemical experiments that the APOBEC3B protein can deaminate the cytosine bases at two sites whose mutant states are subject to high net realized selection intensities—PIK3CA E545K and E542K. By quantifying the effects of mutations, we deepen the molecular understanding of carcinogenesis in head and neck squamous cell carcinoma.

## Introduction

Cancer has long been known to have a basis in somatic mutations that alter a diversity of cellular functions resulting in sustained proliferative signaling, evasion of growth suppressors, and genome instability [1]. Recent high-throughput sequencing of tumors has inspired genomics-driven oncology [2] and enabled the deconvolution of mutations to obtain signatures of their underlying rate [3, 4] and cause [5–7]. However, knowing the source of mutations and the rate at which they occur provides only the first step to understanding the genetic origins of substitutions that drive tumorigenesis and cancer development. After mutations occur, the mutations that are important to cancer cell survival and propagation become prevalent in tumors because of their oncogenic effect on cancer cell lineages. Quantifying the strength of selection of each mutation provides insight into the relative importance of mutations in driver genes [4], while examination of the relative contributions of different mutational processes can reveal the relative importance of each process to tumorigenesis. Independent quantification of mutational processes and selective effects is essential to knowledge-based identification of high value therapeutic targets.

Mutational processes are of particular importance in head and neck squamous cell carcinoma (HNSCC) because of the high proportion of mutations that are attributed to APOBEC (apolipoprotein B mRNA editing catalytic polypeptide-like) enzymes in HNSCC tumors [8, 9]. APOBECs are a family of enzymes that catalyze the deamination of cytosine bases, APOBEC3B expression is higher in cell lines [10] and HNSCC tumors [8] with viral infection, APOBEC activity has been found to be positively correlated with the upregulation of immune signaling pathways [9], and promoter elements and transcription factors linking HPV16 infection and APOBEC3B expression have been identified [11, 12]. Consequently, APOBEC mutagenesis is believed to play a role in the genesis of human papillomavirus-associated (HPV^+^) HNSCC. Some of the earliest insights into the biology of cancer arose as a result of its association with viral infection [13, 14]. Worldwide, an estimated 15 percent of cancers are attributable to viral infection [15], including Epstein-Barr virus, kaposi’s sarcoma-associated herpesvirus, human T-lymphotropic virus type 1, and human papillomavirus (HPV), among others. HPV is responsible for the largest number of viral-related cancers worldwide through its contribution to cervical cancer, anal cancer, vaginal, vulvar, penile cancers, and, increasingly, HNSCC [16].

HPV-associated (HPV^+^) and HPV-unassociated (HPV^−^) HNSCC—related but clearly distinct types of cancer [17, 18]—provide an interesting pairing for comparative analysis of mutational processes and selective effects. Genomic profiling of head and neck squamous cell carcinoma has provided confirmation that, in addition to differences in etiology, natural history and treatment responsiveness [19, 20], HPV^+^ and HPV^−^ HNSCC have distinct biomolecular profiles [21]. Among HPV-associated cancers, the predominant substitutions occurring are activating mutations of *PIK3CA*, inactivating mutations of *TRAF3* or *CYLD*, and amplification of *FGFR3* and the cell cycle gene *E2F*. Among HPV-unrelated cancers, inactivating substitutions in the tumor suppressor genes *TP53* and *CDKN2A* are predominant [17, 21, 22]. These distinctions in epidemiology and molecular biology are complemented by differences in natural history and responsiveness to treatment: HPV-associated tumors are more responsive to cytotoxic and radiation therapy [20], and are more frequently cured [23]. Despite these distinctions between HNSCC caused by tobacco use and those related to HPV infection [24] and a spate of recent de-escalation therapy trials for HPV^+^ HNSCC [25], there are currently no differences in standard therapy specific to these distinct subtypes of HNSCC [26]. Deeper insight into underlying molecular mechanisms may be instrumental to developing more specific therapies.

Patterns within the burden of mutations in a cancer provide insight into the mechanism of mutation [27]. A number of accepted mutational signatures have been identified in HNSCC including two signatures reflective of the activity of APOBEC3 [7, 28, 29]. A previous report suggests that HPV^−^ HNSCC is characterized by a tobacco-associated mutational signature, while HPV^+^ cancers display an APOBEC3 signature and APOBEC3-mediated driver mutations, including characteristic helical domain PIK3CA mutations [8]. However, it is currently unknown whether differences between the genetic architecture of HPV^+^ and HPV^−^ HNSCC can be attributed to distinct underlying mutation frequencies within proto-oncogenes and tumor suppressors, or to differential selection on those mutations. Improved understanding of molecular mechanisms undergirding emergence and maintenance of HNSCC and how the mechanisms differ based on HPV status has the potential to enhance clinical decision-making and inform the development of novel therapeutics. We were interested in understanding the relationship of mutational load to the weight of APOBEC signature, as well as the selective pressure associated with the mutations arising as a consequence of this mutational process.

Next-generation tumor exome sequencing has implicated a number of molecular pathways in HNSCC tumorigenesis. This approach confirmed that known tumor suppressor genes and oncogenes (e.g., *PIK3CA*, *EGFR, CDKN2A*, *NOTCH1, TP53, FBXW7*) play a role in HNSCC, and pathway analyses found that—amongst others—proliferation, differentiation, and PI3K pathways were frequently affected [30–32]. Sequencing-based analyses have expanded our molecular understanding of the disease, and have equated the prevalence of a somatic substitution in tumors of the affected population with its importance in perturbing signaling pathways and tumor development [21, 30, 31]. Identification of targets via prevalence of substitutions assures that the altered gene will be relevant for the affected population, but gives little characterization of the efficacy of targeting the altered gene product at the level of the individual patient. In contrast, quantification of the effects that mutations confer upon the cancer phenotype should assist with prioritization of future precision-targeted therapies [33–35], as well as identify rare substitutions that are significant drivers of tumorigenesis and tumor maintenance.

To characterize these effects, we analyzed a sample of 508 HNSCC tumors from the National Cancer Institute Genomic Data Commons [36] and 17 HNSCC tumors from Hedberg et al [37]. We evaluated the mutational signatures characteristic of HPV^−^ and HPV^+^ HNSCC, quantifying the role that APOBEC plays as a mutagenic factor in HNSCC. We then examined individual substitutions to evaluate the characteristic mutational signature of APOBEC-induced mutations in HNSCC and, noting in particular whether the APOBEC signature is characterized by TCW transition and transversion mutations (wherein the mutated cytosine is underlined), confirmed informatic analysis with in vitro experiment. We analyzed whether APOBEC participates in both HPV^+^ and HPV^−^ mutagenesis and whether an APOBEC signal is correlated with the total mutational load in HPV^−^ and HPV^+^ HNSCC. We then applied an evolutionary biology-based approach to quantify the selection pressure upon specific mutations in a population of HNSCC tumors [4]. Our analysis confirmed well-known HNSCC tumorigenic targets, but also highlights low-prevalence mutations that are major drivers in a minority of patients, and implicates novel targets as potential drivers in HNSCC development. We quantified the importance of APOBEC mutagenic processes, identifying the underlying mutation frequencies of molecular variants and the selective effects on them that lead to the development of HNSCC.

## Results

### HPV^−^ and HPV^+^ classification and HPV viral load

Our analysis incorporated 69 HPV^+^ and 451 HPV^–^ cases (Supplementary Table S1) retrieved from National Cancer Institute Genomic Data Commons [36] and from tumor-normal pairs from the University of Pittsburgh head and neck tissue bank, reported in Hedberg et al. [37], and sequenced at the Yale Center for Genome Analysis. Of the 69 HPV^+^ HNSCC, one tumor carried a *TP53* substitution (missense, in this case)—a tumor that was also classified as HPV^+^ in the comprehensive genomic characterization of HNSCC conducted by TCGA (TCGA-CR-7638) [21]. This rarity is consistent with a previously described model wherein HPV-driven cancer is associated with high HPV viral load, wild type p53, and overexpression of p16 as a consequence of HPV oncoproteins that inactivate p53 and Rb. Non-HPV-driven cancer frequently exhibited *TP53* substitutions and an absence of p16 expression, although in some cases HPV was present at low viral load [38]. Our HPV designations differ from the TCGA biospecimen supplement data designations that are based on PCR-based MassArray—a method that has been deemed too sensitive to accurately determine whether HPV infection has played a causal role in tumorigenesis [21]. Indeed, 93 out of 508 TCGA HNSCC tumors had detectable portions of the HPV genome using this method, and 24 of these 93 tumors had substitutions in *TP53*, meaning they were unlikely to be driven by viral oncogenesis due to the criteria outlined above; thus, we applied an alternate method of detecting viral sequences in RNA-Seq data that has been relied upon in previous studies [21, 39]. Among TCGA samples with available HPV status and available mRNA expression data (429 HPV^−^ and 66 HPV^+^ tumors, mRNA expression data obtained from cBioPortal [40, 41]), mRNA expression was higher for APOBEC3B, APOBEC3C, APOBEC3D, APOBEC3F, APOBEC3G, and APOBEC3H in HPV^+^ vs HPV^−^ tumors (*P* < 0.001, one-sided Welch’s *t* tests), and APOBEC3A mRNA expression levels were not significantly different between the two HPV classifications (*P* = 0.38, one-sided Welch’s *t* test; Figure S1), as previously reported on a smaller subset of HNSC tumors [8]. Within the germline TCGA data, the distribution of the global APOBEC3H haplotypes [42] are 27.6% haplotype 1, 17.7% haplotype 2, 11.8% haplotype 3, 42.3% haplotype 4, and 0.6% other [43].

### Between HPV^+^ and HPV^−^ tumor tissues, signatures of mutagenesis are not distinct, but frequencies of mutation among genes are distinct

**Fig. 1:**
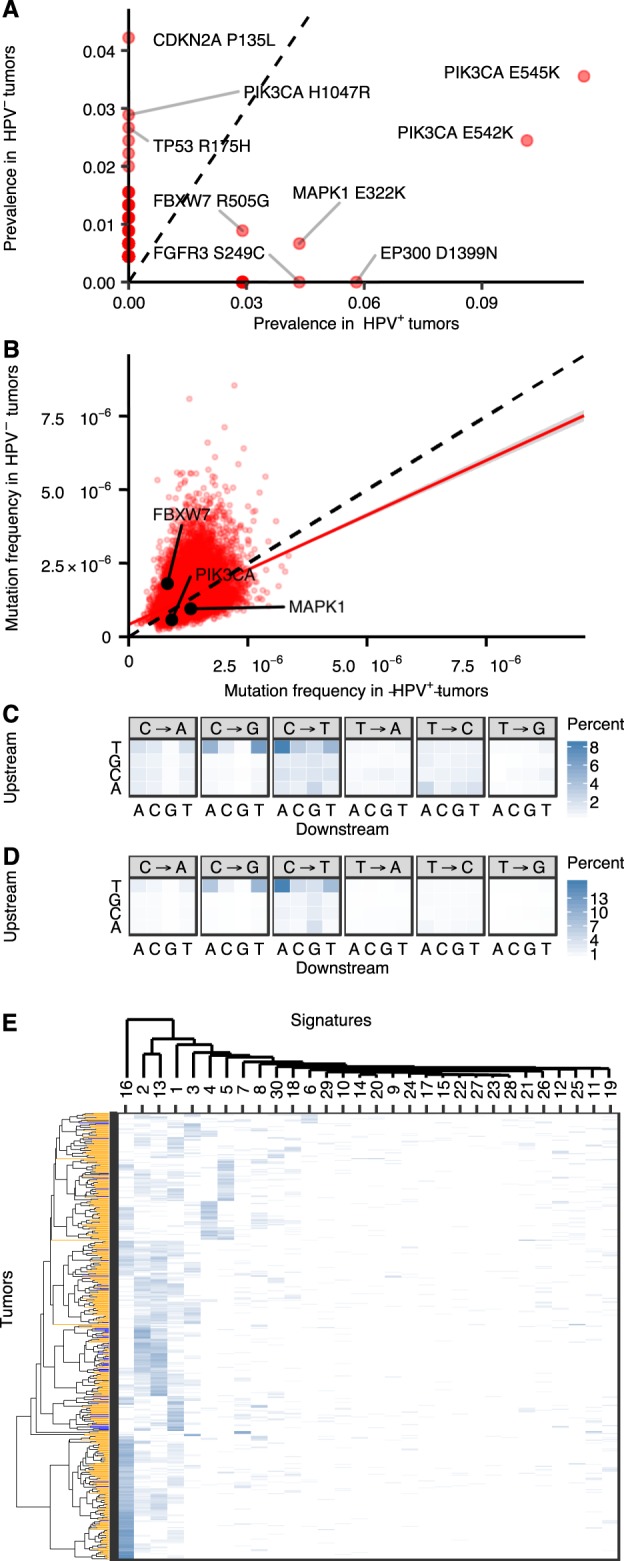
Prevalences of recurrent substitutions and expected mutation frequencies. **a** Prevalences of recurrent substitutions in HPV^−^ and HPV^+^ HNSCC tumor tissues. Labels convey the HUGO gene name and the amino acid change (*: STOP codon) and the dashed line depicts *y* *=* *x*. **b** Gene-level mutation frequencies among HPV^−^ and HPV^+^. The black line depicts *y* *=* *x*, and the red line depicts the result of a linear regression that achieves high statistical significance but extremely poor fit to the data (*P* < 10^−16^, *R*^2^ = 0.01). Three shared recurrently mutated genes are labeled in black. Within genes, trinucleotide mutation frequencies inferred from **c** 451 HPV^−^ HNSCCs and **d** 69 HPV^+^ HNSCCs are similar. Percentage of mutations of each trinucleotide type is reported numerically in each cell (white: low to dark blue: high). **e** Heat map of the 30 COSMIC mutational signatures within 461 HNSCC tumor exome sequences. HPV^+^ tumors (blue), HPV^−^ tumors (yellow), and tumors with unknown HPV status (black) are structured by their mutational signatures using hierarchical agglomerative complete linkage clustering

To probe into the relative importance of the substitutions driving these two types of HNSCC, we examined the prevalence of substitutions among the cancers, the frequency we expected to observe substitutions in the absence of selection, and ultimately the intensity of selection that drives any difference between the observed and expected fluxes. The prevalences of recurrent somatic nucleotide variants in HPV^+^ and HPV^−^ tumor tissue were markedly different: only four substitutions were recurrently mutated in the assembled data sets for these cancers (Fig. 1a, Supplementary Table S2). Some of these differences in prevalence arise because of differences in gene-level mutation frequencies (Fig. 1b, Supplementary Table S3) that reflecting underlying differences between the covariates governing gene-level mutation events—such as gene expression levels—within HPV^+^ and HPV^–^ cells [21]. In contrast, differences in prevalence between HPV^+^ and HPV^−^ tumors are unlikely to arise because of differing trinucleotide mutation frequencies, because HPV^+^ and HPV^−^ tumor tissues exhibited similar trinucleotide mutation profiles (Figs. 1c, d). The dominant signatures in these trinucleotide mutation profiles include APOBEC-related C → T and C → G mutations in the TCW motif context, and C → T mutations in a 5′-CG-3′ context that are likely reflective of spontaneous deamination of 5-methylcytosine and correlated with aging (Signature 1 of 30) [6].

### APOBEC signatures are not equally frequent in HPV^−^ and HPV^+^ HNSCC

All 30 COSMIC mutational signatures were represented in one or more of the 461 HNSCC primary tumors examined that exhibited greater than 50 mutations (Fig. 1e, Supplementary Table S4). The two most common signatures were those attributed to APOBEC activity (COSMIC signatures #2 and #13). After APOBEC, signature #1, a typical somatic mutation signature attributed to aging, was the most prevalent signature in HNSCC—higher than tobacco (#4). Mean weights of signatures #13 and #2 were 0.19 and 0.30 in HPV^+^ tumors and 0.13 and 0.11 in HPV^−^ tumors, respectively.

**Fig. 2:**
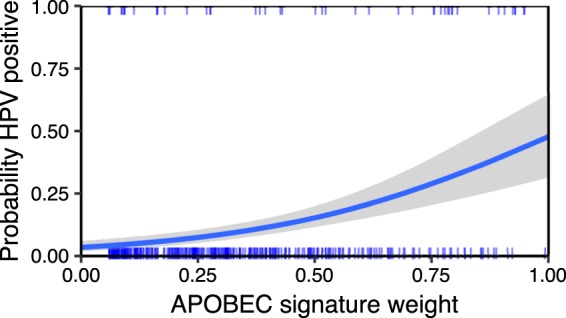
Tumors with a high total APOBEC weight were more likely to be HPV positive

APOBEC signatures were not equally frequent in HPV^−^ and HPV^+^ HNSCC (Fisher’s exact test, *P* = 0.0001). Among tumors with a known HPV status and enough SNVs to calculate mutational signatures [5], 98% of HPV^+^ HNSCC tumors exhibited an APOBEC signature, while 76% of HPV^−^ tumors exhibited an APOBEC signature. Consistent with expectation, HPV^+^ status in tumor tissue was more likely among those tumors exhibiting a high total APOBEC weight (Fig. 2; logistic regression, *P* = 1 × 10^−8^).

### HPV^+^ and HPV^−^ tumors with a signature of APOBEC-associated mutagenesis do not differ in mutation load, but do differ in the proportion of TCW→TKW substitutions

In HPV^−^ tumors, there was no significant difference between the typical SNV count of tumors with and without an APOBEC signature (*P* = 0.49, two-sided Wilcoxon rank-sum test). Only one tumor was both HPV^+^ and had no detectable signature of APOBEC activity (PY-16T). Among samples with an APOBEC signal, typical mutation load was not significantly different between HPV^−^ and HPV^+^ HNSCC (*P* = 0.39, two-sided Wilcoxon rank-sum test). Among tumors with an APOBEC signature, HPV^+^ samples typically exhibited a higher proportion of the TCW to TKW mutations that are commonly attributed to APOBEC (*P* = 0.008, two-sided Wilcoxon rank-sum test). Nevertheless, the majority of TCW to TKW mutations among tumors with an APOBEC signal occurred within HPV^−^ tumors, as a supermajority of tumors were HPV^−^. Thus, when analyzing the substitutions that likely occurred through APOBEC mutagenesis, we consider all HNSCC samples regardless of HPV status.

### The selective pressures within HPV^+^ and HPV^−^ HNSCC tumors largely differ

**Fig. 3:**
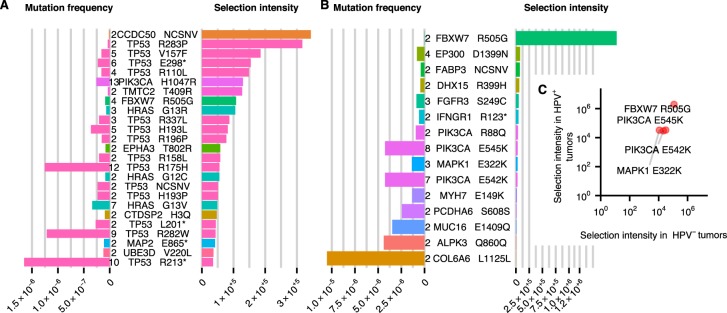
Expected mutation frequency (left-hand bar), prevalence (columnated numbers, along with HUGO gene name and the amino acid change), and selection intensity (right-hand bar) associated with recurrently observed mutations (**a**) for the top 25 selection intensities of point mutations among 451 HPV^−^ HNSCC tumor tissues, and (**b**) for all recurrent substitutions among 69 HPV^+^ HNSCC tumor tissues. Inset, (**c**) the selection intensities on the recurrently observed mutations within HPV^−^ HNSCCs and within HPV^+^ HNSCCs

Much of the difference in the frequencies of substitutions between HPV^+^ and HPV^–^ HNSCC can be attributed to different selective intensities imposed by mutations occurring in the HPV^+^ vs. HPV^–^ context. Comparison of observed frequencies of substitutions and the expected frequencies of mutations yielded a ranked list of single nucleotide variants based on their selection intensity (Fig. 3, Supplementary Table S2), excluding variants observed fewer than two times in our sample set. These recurrent variants represent the point mutations that are the strongest drivers of the neoplastic HNSCC disease phenotype. Among the recurrent SNV with the largest 25 selection intensities in HPV^−^ HNSCC and all recurrent SNV in HPV^+^, only FBXW7 R505G was shared. Substitutions in TP53 and HRAS are frequent in HPV^−^, but are almost never found in HPV^+^. Among all variants, four recurrent substitutions are shared (MAPK1 E322K, PIK3CA E542K and E545K, and FBXW7 R505G) and have similar selection intensities within the two tumor types (Fig. 3c).

Within this set of the strongest drivers, many align with existing understanding of HNSCC tumorigenesis. Several of the most-selected variants occurred in genes—*TP53*, *HRAS*, *PIK3CA, MAPK1*—previously associated with the landscape of substitutions observed in HNSCC via genomic screening [17, 31]. Interestingly, one of the most strongly selected variants among HPV^−^ HNSCC (and the strongest selected variant in HPV^+^ HNSCCs) is characterized by a low expected mutation frequency and a low prevalence of oncogenic substitution, occurring in the E3 ubiquitin ligase-encoding gene *FBXW7*, a known tumor suppressor which has been molecularly characterized as a regulator of common HNSCC oncogenes [44, 45]. In HPV^+^ tumors, fibroblast growth factor receptor genes such as FGFR3 have previously been found to be enriched for mutations [17], but the high intensity of their effect on the proliferation and survival of cancer cell lineages has not previously been quantified relative to other somatic nucleotide mutations.

Other variants with high inferred intensity of selection implicate novel candidate genes in HNSCC tumorigenesis or cancer development also deserve further attention to validate their role. The coiled-coil domain-containing protein 50 (*CCDC50*) gene contained a recurrent splice-site substitution within two HPV^–^ tumors: this mutation was found to have the highest selection intensity of all recurrent somatic nucleotide variants within HPV^–^ tumors, as it is subject to an extremely low expected frequency of mutation. The four lowest selection intensities among recurrently observed mutations in HPV^+^ tumors were one substitution within the second longest human protein MUC16 (the *MUC16* gene is subject to frequent mutation in cancers and is likely spuriously associated with tumorigenesis [3]), and three synonymous substitutions within *ALPK3*, *PCDHA6*, and *COL6A6*.

### Substitutions from the putative APOBEC binding site have a lower average selection intensity

**Fig. 4:**
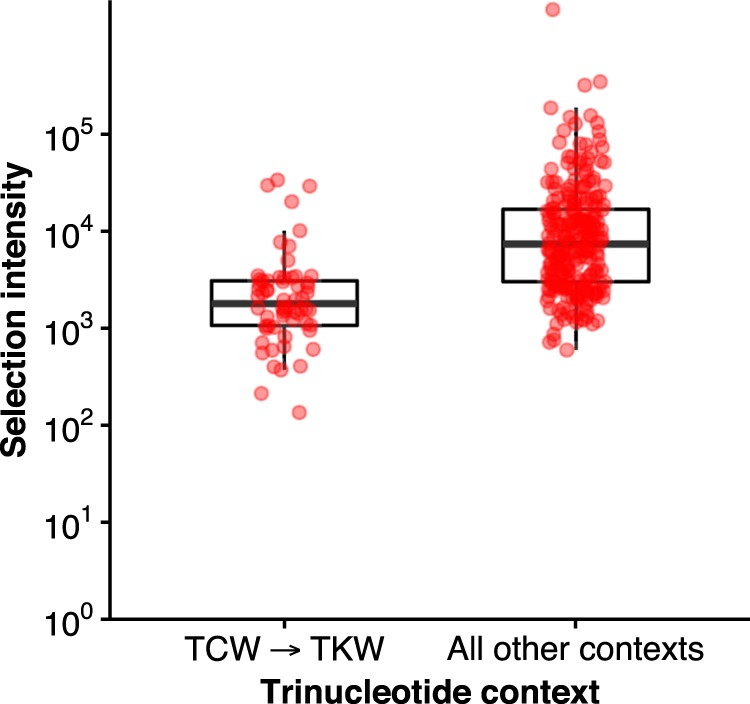
The selection intensities of recurrent, amino acid replacement TCW→TKW nucleotide mutations compared to other recurrent, amino acid replacement mutations (All other contexts)

Among the 314 recurrent amino acid substitutions in the HPV^+^ and HPV^–^ datasets, 62 were the product of a TCW to TKW nucleotide mutation, and thus can be putatively associated with APOBEC mutagenesis. Substitutions that could putatively be attributed to APOBEC mutagenesis typically exhibited a lower selection intensity than substitutions that are not in a TCW to TKW trinucleotide context (Fig. 4; Supplementary Table S2, *P* = 0.002, one-sided Welch two-sample *t*-test).

### *PIK3CA*, FBXW7, and FGFR3 are the genes whose APOBEC-based tumor burden contributes the most to population-level cancer

**Fig. 5:**
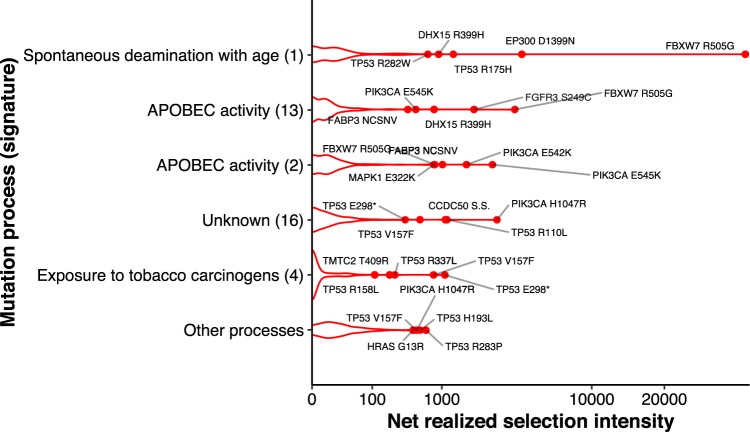
Net realized selection intensity within cancer patients for recurrently observed mutations attributable to signatures 1, 2, 13, 4, 16, and the remaining 25 signatures. PABP3 NCSNV is a non-coding single-nucleotide variant on chromosome one at position 31838696

Next, we quantified tumor burden arising from APOBEC processes by evaluating which mutations have the largest effect on cancer growth and the highest prevalences among tumors, and distinguished whether these mutations most likely result from APOBEC mutagenesis. Among mutations that were likely APOBEC-independent, FBXW7 R505G, EP300 D1399N, PIK3CA H1047R, and several mutations in TP53 had the highest net realized selection intensity. Among mutations that were likely APOBEC induced, FBXW7 R505G, PIK3CA E545K, FGFR3 S249C, PIK3CA E542K, and DHX15 R399H had the highest net realized selection intensities. (Fig. 5; Supplementary Table S5).

### APOBEC3B efficiently deaminates PIK3CA DNA in vitro

**Fig. 6:**
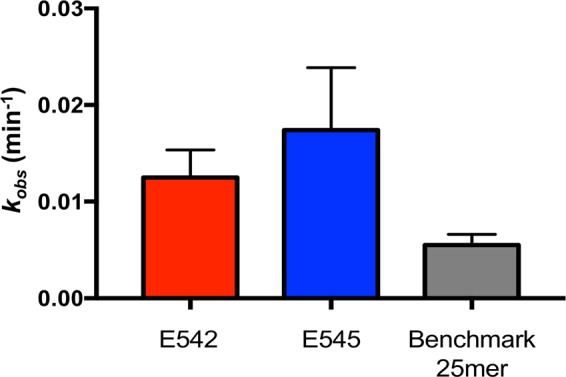
Deamination of *PIK3CA* mimic substrates. UDG deamination experiments were conducted with 25-mer substrates corresponding to the *PIK3CA* E542 site, E545 site, and the benchmark 25-mer. The bar graph shows a comparison of the observed catalytic rate *k*_obs_ with each oligonucleotide substrate. Error bars represent the standard deviation of the exponential fit to the data

To validate whether these bioinformatically identified targets are true APOBEC targets, we investigated deamination of sites in *PIK3CA* using a traditional uracil DNA glycosylase (UDG) deaminase assay. Using a recombinantly expressed and purified preparation of full-length APOBEC3B—the APOBEC3 member most closely associated to human cancers—we tested its activity on two 25-mer substrates mimicking the DNA sequence surrounding the E542 and E545 sites. APOBEC3 deaminated the TC motif in the center of these two sequences more than it deaminated a centered TC motif within an AT-rich 25-mer non-specific oligonucleotide control. Upon determination of catalytic rates by fitting of the deamination time course to a single exponential curve, we found that both *PIK3CA*-derived oligonucleotides were deaminated at a higher efficiency than the benchmark 25-mer, which we adapted from a published 43-mer [46], reflecting published literature including a systematic biochemical analyses of all 16 cytosine-flanking trinucleotide studies and structural studies that captured thymine at the −1 position relative to the target cytosine [47] (Fig. 6). This experiment shows that A3B can deaminate single-stranded DNA matching these cancer mutation hotspots.

## Discussion

Here we have shown that the COSMIC mutation signatures of HPV^+^ and HPV^−^ head and neck squamous cell carcinomas do not cluster into distinct groups of self-similar mutation signatures when considering all 30 COSMIC signatures. Nonetheless, all but one (98%) HPV^+^ tumor had a detectable APOBEC signature, whereas a considerably lower percentage (76%) of HPV^−^ tumors had a detectable APOBEC signature. Our analysis revealed significant differences in the genetic architecture of HPV^+^ and HPV^–^ HNSCC tumors. Evaluating each mutation based on its expected frequency in the absence of positive selection and the actual frequency at which it was observed, we identified several well-known culprits: *PIK3CA, TP53*, and *HRAS* [21]. Our analysis of the effect sizes of mutations underlying HPV^+^ and HPV^−^ HNSCC tumors not only substantiates the difference in genetic architecture between these tumors based on HPV status, but also identifies some rare oncogenic mutations that contribute strongly to tumorigenesis in both HPV^+^ and HPV^−^ HNSCC tumors (e.g., FBXW7 R505G), as well as oncogenic mutations that are somatically selected for in either HPV^+^ (e.g., EP300 D1399N, FGFR3 F249C) or HPV^−^ tumors (e.g., PIK3CA H1047R, oncogenic mutations in HRAS and TP53). Our results primarily agree with the existing literature on the strongest driver mutations in HNSCC, but segregate the contributions of oncogenes in particular as major contributors to one or the other of HPV^+^ and HPV^–^ HNSCC tumors.

It has been previously remarked that mutations arising from APOBEC-related processes could be a double-edged sword: [48] while APOBEC-related processes provide innate immunity against viruses by targeting them for mutation [49], they also are a source of numerous mutations driving cancer [7, 28, 50–52]. We have shown that the inner edge of that sword is somewhat blunt: APOBEC mutations typically have lower selection intensities than mutations caused by non-APOBEC processes. Our analysis implies that it would on average be preferable during tumorigenesis for a person to exchange an unknown, non-APOBEC neoplastic mutation for an unknown, putatively APOBEC neoplastic mutation. Regardless, it remains highly plausible that prevention of APOBEC mutagenesis during tumorigenesis would diminish the rate of substitution and decelerate cancer progression and even the evolution of therapeutic resistance [53]. Indeed, some of the variants with the highest net realized selection intensities among patients are attributable to APOBEC activity. The net realized selection intensity of mutations quantifies the amount of cancer phenotype at the population level that is attributable to their cause, incorporating both the prevalence of each substitution and the degree to which it contributes to the cancer phenotype in the patients with that substitution. Accordingly, it quantifies the population-wide amount of cancer phenotype that could be eliminated by universal patient prescription of a perfect therapeutic that abrogates oncogenic function of each mutation, corresponding to potential population health benefits of a perfect mutation-targeted therapeutic. One caveat of our analysis is that this inference applies to a population as though our data were a representative sample of the population. HPV infection prevalence varies with socioeconomic factors [54], racial disparities [35, 55] and genetic polymorphisms [56, 57] associated with outcomes in head and neck cancer so greater attention to representative sampling would provide more precise estimates of the frequencies of mutations within populations [58] and the net realized selection intensities in each population.

The mutations in *PIK3CA* that are highly selected in HPV^+^ HNSCC—E542K and E545K—lie in its helical domain [59]. Mutated sites in the helical domain have been proposed as targets of APOBEC-mediated cytosine deamination [8]. The Henderson et al. (2014) analysis reported these helical domain substitutions to be HPV^+^ HNSCC-specific based on correlations between APOBEC3B expression, and HPV status and the prevalence of TCW→TKW substitutions, yet it appears that the cancer effect size of these mutations is substantial in both HPV^–^ HNSCC and HPV^+^ HNSCC. The four shared recurrently observed mutations among HPV^–^ and HPV^+^ HNSCC are similarly ordered by selection intensity within their tumor type.

Our biochemical experimental data show that APOBEC3B deaminates the two PIK3CA mimic oligonucleotides at a higher efficiency compared to a benchmark oligomer of a similar size. Importantly, this analysis is the first quantitative assessment of deamination of two bioinformatically identified putative target sites relative to a benchmark using a full-length APOBEC enzyme. This observation is particularly interesting given that we are beginning to understand the molecular features of APOBEC-driven mutagenesis in vivo. For instance, APOBEC mutation patterns are present at a significant levels in six distinct cancer types [28] and APOBEC proteins specifically target regions of the human genome containing exposed single stranded DNA, including chromosome rearrangement breakpoints [51] and replication intermediates [60]. This specificity of targeting might explain observations of a temporal dependence within the cell cycle, wherein APOBEC specifically induces mutations in early replicating regions in chromosomal DNA that are highly active in gene expression [52]. In light of these bioinformatic discoveries, our biochemical result demonstrating context-specific targeting of APOBEC3B provides an additional layer of validation of APOBEC mutagenesis in cancer, and opens new questions in the field—including questions regarding the molecular mechanisms by which APOBEC enzymes target these sites in specific genes.

A key difference in our methodology from the gene-level focus of other recent exome sequencing studies is the application of inference at the level of individual mutations of amino acid residues in our analysis. Our analysis is therefore restricted to substitution mutations, whereas comprehensive genomic characterizations have investigated deletions, copy number alterations, and other rare genomic events. For instance, Hajek et al [22] flagged deletions in the gene *TRAF3*, demonstrating the importance of nonsense mutations in this gene to the genetic architecture of HPV^+^ HNSCC. Because our approach enables the estimation of expected frequencies of nucleotide mutations, but not expected frequencies of deletions, the lack of recurrent *TRAF3* substitutions precluded it from any estimation of selective pressure using our current methodology.

In addition to refining the interpretation of genes exhibiting higher-than-expected mutation loads, our analysis suggests some novel avenues for investigation. This methodology highlights gene variants that have been missed by gene-based surveys of somatic substitution prevalence. One of the somatic variants with very high effect sizes in both HPV^+^ and HPV^−^ tumors, FBXW7 R505G, occurs in an E3 ubiquitin ligase and modifies E3 ligase binding to the substrates [61, 62]. *FBXW7* has been characterized as a tumor suppressor due to its ability to target several known oncoproteins including MYC, NOTCH, and Cyclin E [44]. FBXW7 is also a negative regulator of Aurora A and B, and thus regulates a mitotic checkpoint [63, 64]. *FBXW7* mutation has been demonstrated to associate with NOTCH1 activation in T-cell acute lymphoblastic leukemia; [65] however, not until *NOTCH1*’s implication in HNSCC tumorigenesis was *FBXW7* linked to this disease [30, 45].

These results underscore one important difference of our evolutionary approach from earlier approaches toward the identification of the molecular basis of tumorigenesis and cancer development from exome sequence data: its utility at ranking oncogenic mutations by importance, making use of tissue- and nucleotide- specific mutation frequencies. Here, we make use of bulk sequencing of primary tumors at the time of resection, and thus our estimates are based on average mutation frequencies and correspond to selection intensities that are averaged over the time course of tumorigenesis to resection. However, processes of mutagenesis vary over time [66], and finer resolution measurements of the expected frequencies of mutations [67] and corresponding selection intensities are possible, especially with multiple samples gathered metachronously or even synchronously. Our approach has been enabled by extensive previous research on the intrinsic mutation rates of genes and their covariates [3, 68, 69], and relative mutation rates of single nucleotide variants and their putative mutagenic source [7]. Nonetheless, comparisons of selection intensities on somatic single nucleotide variants only paint part of the complex picture of tumorigenesis: copy number variation, epigenetic alterations, tumor heterogeneity and microenvironment, germline variants, and other factors also contribute to the cancer phenotype [58, 70–72]. Methods to calculate measures of selection intensities that incorporate these factors and their epistatic effects would enable a holistic model—and a more holistic understanding—of tumorigenesis.

## Methods

### Determining HPV status

Curated sequencing data for 508 cases of HNSCC was sourced from National Cancer Institute Genomic Data Commons (NCI GDC) [36], which provides information on the limited diversity of ethnicity and race in the sample. 477 of these tumors had HPV RNA viral transcript loads as assessed by the VirusScan software in Cao et al [39]. Out of the 31 tumors with no previous viral load data, 24 had the RNA-Seq data required for VirusScan. HPV infection status for these data was determined by counting HPV16 and HPV33 reads within RNA-Seq data, as attributed by VirusScan [39]. Tumors were designated as HPV^+^ if they contained greater than 100 HPV RNA viral transcript reads per hundred million, and tumors were designated HPV^–^ if they contained fewer than 100 HPV RNA viral transcript reads per hundred million. Of the 501 HNSCC tumors analyzed with VirusScan, 66 tumors were deemed HPV^+^, and 435 HNSCCs that did not match that criterion were classified HPV^–^. Out of the remaining seven tumors sourced from the NCI GDC with no viral load data, two tumors had consistent clinical results from p16 and ISH tests (one HPV^+^ and one HPV^–^). Additionally, we included 17 HNSCCs that were whole-exome sequenced at Yale University [37] and one (PY-16T) was also determined to be HPV-associated by HPV in situ hybridization and p16 immunohistochemistry [73], and the remaining 16 cases were determined to be HPV^–^.

### Bioinformatic and statistical analysis of HNSCC data

Before analysis, all SNV data downloaded from the NCI were converted to human genome 19 reference coordinates using the R package rtracklayer [74] to make the data compatible with the various software packages used in our analysis. Somatic variants from the Hedberg et al [37]. dataset were obtained from published supplementary material, and underwent the following processing: (1) variants with labels other than “Transmitted” and “Primary Only” were removed to obtain the set of primary tumor variants, (2) neighboring SNVs were redesignated as DNVs and extraneous alleles (denoted with lower case) were removed, (3) variants were re-annotated using the software vcf2maf v1.6.13 [75, 76], (4) those variants annotated as “common” or classified as intronic and flanking were removed, and (5) shared variants for five tumors that were identified as duplicated in the NCI dataset (PY-19T, PY-1T, PY-14T, PY-13T, PY-7T) were removed. For the selection intensity analysis, we removed all neighboring variants to ensure that DNVs did not influence our analyses of the frequencies of single nucleotide mutations. We performed hierarchical clustering on the distance matrix of mutational signatures for all tumors that had available data on HPV status and mutational signatures.

### Calculating selection intensity

The selection intensity for point mutations was calculated by an updated approach based on that of Cannataro et al [4]. Briefly, the expected frequency *μ* that nucleotide mutations occur before they are acted on by selection over the average amount of time elapsed throughout the evolutionary process driving tumorigenesis (from initialization to resection) was determined by calculating the expected frequency that silent mutations occur at the gene level using dndscv [91], then scaling each possible nucleotide mutation by a coefficient corresponding to the relative expected frequency within its trinucleotide context. The effect of the trinucleotide context was quantified as the average distribution of trinucleotide contexts for that tissue calculated analyzing all HNSCC tumors with greater than 50 mutations with deconstructSigs [5]. We then defined the frequency of substitution, *λ*, as the frequency at which genetic variants were observed within sequence data. We corrected for the fact that one can only observe one substitution per site, even though a flux of mutations at a given rate will generate a Poisson distributed number of substitutions [4]. This observed frequency of substitutions was divided by the expected frequency of mutations to determine $\frac{\lambda }{\mu } = \gamma$, the selection intensity on a point mutation during the intratumoral fixation process.

### Calculating net realized selection intensity per putative mutation process

We calculated the effect sizes *γ*_*i*_ of somatic amino acid substitution *i* within a growing tumor. Effect sizes were calculated using the prevalence *p*_*i*,h_ of that substitution among tumors, where $p_{i,h} = \frac{{n_{i,h}}}{{N_h}}$, *n*_*i,h*_ is the number of tumors with variant *i* detected in tumors with HPV status *h*, *N*_*h*_ is the total number of tumors with HPV status *h*$\in \{ 0,1\}$, where *h* = 0 indicates HPV^–^ and *h* = 1 indicates HPV^+^). The net realized selection intensity was then $\frac{{p_{i,h_1}\left( {\alpha _{i,j}\gamma _ip_{i,h_1}} \right) + p_{i,h_2}\left( {\alpha _{i,j}\gamma _ip_{i,h_2}} \right)}}{{p_{i,h_1} + p_{i,h_2}}}$ for all recurrent amino acid substitutions *i* in our dataset, where the median proportional contribution of the individual COSMIC signatures to the specific single nucleotide substitution *j* resulting in amino acid substitution *i* was *α*_*i,j*_.

### Code availability

The scripts used to process data and perform these analyses are available online at https://github.com/Townsend-Lab-Yale/HNSCC_APOBEC.

### Assessing APOBEC3B deamination of PIK3CA helical domain sites

Recombinant full-length APOBEC3B with a N-terminal maltose binding protein tag was sequenced to ensure that the plasmid DNA did not contain any insertions, deletions, or mutations. The plasmid was expressed and purified from *E. coli* (the Overexpress C43(DE3) cell line purchased from Lucigen) as described previously [77] and authenticated using antibiotic selection markers. For deamination assays, a traditional UDG deaminase assay was employed in which 1 µM of APOBEC3B was incubated with 40 nM of 5′-radiolabeled oligonucleotide in a reaction buffer consisting of 50 mM Tris pH 8.0, 100 mM NaCl, 0.1% Triton X-100, and 1 mM DTT. Upon quenching timepoints, the reactions were post-processed with 5 units of UDG and 0.2 N NaOH. The resulting solution was then run on a 20% denaturing PAGE gel and exposed to a phosphor screen overnight. Gel densities were analyzed with QuantityOne software to calculate % product at each timepoint. A representative gel image from which the densitometry analysis has been performed is available as a supplement to the manuscript (Figure S2). Catalytic efficiency of the purified full length APOBEC3B protein has been reported in a number of previous publications [77, 78]. While differences exist in constructs including expression system, presence of affinity/solubility tags, length of oligonucleotide substrate, and assay conditions used, the APOBEC3B activity in the present study reflects expected values based on previously reported kinetics. The benchmark oligonucleotide tested in the current study is a shortened version (25-mer) generic oligonucleotide substrate based on early characterization of APOBEC3B [46]. Our studies also confirm that catalytic efficiency increases proportionally to substrate length (data not shown) similar to that previously shown with APOBEC3G [79].

All DNA oligonucleotides were ordered HPLC-purified from Integrative DNA Technologies. The E542 and E545 sites are located very close to one another in PI3KCA; thus, when we constructed the 25-mer PI3KCA test oligos, the sequences had significant overlap. To ensure that our analysis focused on the deamination of each respective cytosine, we constructed oligos for each site that were composed with a thymine (another pyrimidine) at the other site. The test oligonucleotide sequences,

E542: 5′-TGCTTAGTGATTTCAGAGAGAGGAT-3′, and

E545: 5′-TGCTCAGTGATTTTAGAGAGAGGAT-3′, were based on the cDNA sequence of PIK3CA surrounding the test sites. The benchmark 25-mer oligonucleotide sequence

control: 5′-ATTATTATGGATCAATTATTTATTA-3′,

was a modification of a substrate oligonucleotide used in an earlier report using this assay to investigate activity of APOBEC3B [46].

For calculation of the observed catalytic rate *k*_obs_, each enzymatic time course was fit to a single exponential curve defined by

$$\% {\mathrm{product = }}A(1 - e^{ - k_{obs}t})$$

in which *A* is the maximum % product, and time *t* was measured in minutes.

## Supplementary information

Supplementary Figure S1

Supplementary Figure S2

Supplementary Table S1

Supplementary Table S2

Supplementary Table S3

Supplementary Table S4

Supplementary Table S5
