# Supplementary figures and images for: APOBEC-induced mutations and their cancer effect size in head and neck squamous cell carcinoma
Source: Oncogene. 2019 Jan 15;38(18):3475–87. doi: 10.1038/s41388-018-0657-6 (PMC6499643; doi:10.1038/s41388-018-0657-6)

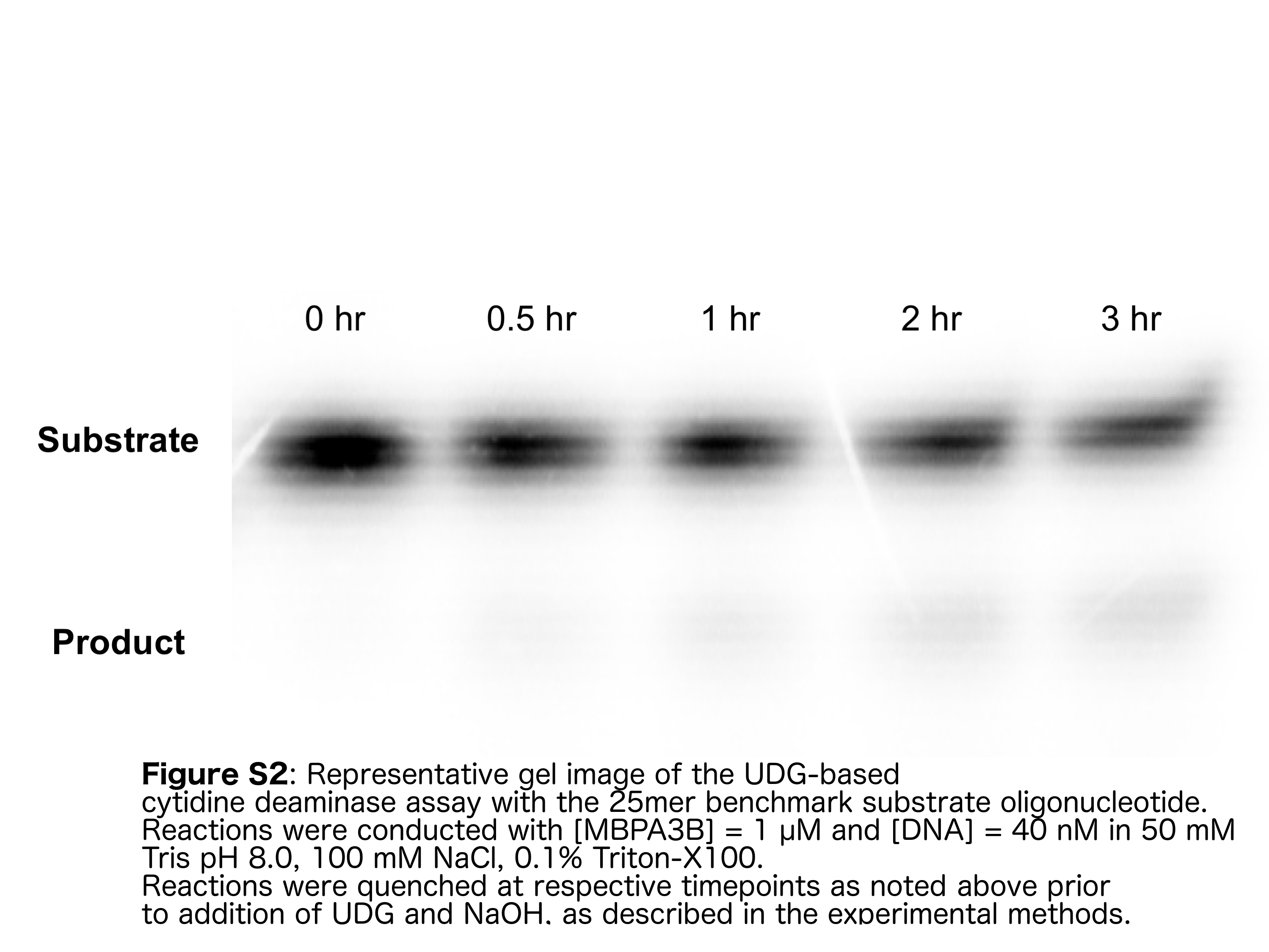

Supplement: Supplementary file 2 — Supplementary Figure S2 [file 41388_2018_657_MOESM2_ESM.tif]
